# Mercury distribution in the great cormorant (*Phalacrocorax carbo*) from the Krogulna ponds and Nysa Kłodzka River (Poland)

**DOI:** 10.17221/16/2023-VETMED

**Published:** 2023-04-25

**Authors:** Kamila Novotna Kruzikova, Zuzana Siroka, Tomas Kral, Piotr Hliwa, Piotr Gomulka, Anna Spodniewska, Zdenka Svobodova

**Affiliations:** ^1^Department of Animal Protection and Welfare and Veterinary Public Health, Faculty of Veterinary Hygiene and Ecology, University of Veterinary Sciences Brno, Brno, Czech Republic; ^2^Department of Ichtyology and Aquaculture, Faculty of Animal Bioengineering, University of Warmia and Mazury in Olsztyn, Olsztyn, Poland; ^3^Department of Pharmacology and Toxicology, Faculty of Veterinary Medicine, University of Warmia and Mazury in Olsztyn, Olsztyn, Poland

**Keywords:** heavy metal, kidney, liver, mining, muscle, piscivores

## Abstract

Mercury belongs among the highly hazardous substances present in the environment and represents a great health risk for many animals including predatory and piscivorous birds. The aim of this study was to assess the total mercury content in the main detoxifying organs (liver and kidney) and in the muscles of adult great cormorants (*Phalacrocorax carbo*) caught at two localities (the Krogulna ponds and the Nysa Kłodzka River) in southern Poland. The aim was to compare the locality with the iron ore mining history (the Krogulna ponds) with a site without such load (the Nysa Kłodzka River). The total mercury content in the great cormorants decreases as follows: kidneys > liver > muscle in both monitored localities and significantly differs between the localities. The average mercury content varied from 0.58 ± 0.38 mg/kg (muscle) to 1.39 ± 1.42 mg/kg (kidney) in the cormorant from the Krogulna ponds and from 1.09 ± 0.40 mg/kg (muscle) to 3.12 ± 1.55 mg/kg (kidney) in the cormorant from the Nysa Kłodzka River. The accumulation of mercury does not correlate with the mining history as it was higher at the Nysa Kłodzka River, but it is probably influenced by the different fish stocks in these two localities (omnivorous fish in the Krogulna ponds versus predatory fish in the Nysa Kłodzka River).

According to the Agency for Toxic Substances and Disease Registry (ATSDR) and also according to the World Health Organization (WHO), mercury belongs among the ten most hazardous substances present in the environment and is dangerous for one’s health (Wang 2012). It is a naturally occurring element that is released from the Earth’s crust into the environment by numerous activities (small-scale gold extraction, burning fossil fuels, production of synthetic chemicals, volcanic activity, etc.) (Marnane 2018).

Although all forms of mercury show toxic effects, the exact toxic impact of Hg on organisms, mainly animals and humans, depends on its chemical form, dose and route of exposure. Absorption into the animal body is possible via inhalation or ingestion, the main sources being water and food. Skin exposure is not considered important. Inhalation is also not the main route of exposure in European mammals and most other animals, though the contamination of soil, the aquatic environment and consequently the food chain is closely connected to mercury pollution in the air, as around 50% of the mercury deposited annually in Europe originates from outside Europe and undergoes long-distance transport, the global mercury cycle (Marnane 2018). On the other hand, animals such as migratory birds, spending long times in air masses, might also be possibly exposed to mercury poisoning via inhalation.

Mercury found as a consequence of natural and anthropogenic contamination in rivers, lakes and oceans represents a great health risk for many animals including predatory and piscivorous birds. Elemental mercury and its inorganic compounds are still used in industry and are discharged via waste waters into the aquatic environment where they can be transformed by sediment microbiota into the highly toxic organic form, methylmercury. Its creation may be dependent on several factors, e.g., the temperature, which influences the microbial enzymatic activity, and thus differs regionally (Kaler et al. 2014). This form is lipophilic, easily absorbed by higher organisms and rapidly incorporated into the food chain where it is accumulated and finally biomagnified in predators (Klapstein and O’Driscoll 2018).

Both acute and chronic exposure to mercury and its compounds result in health damage. Central nervous system defects, kidney damage as well as cardiomyopathy and arrhythmia belong among the most important damage to health caused by exposure to mercury. Young individuals and embryos are extremely sensitive to this element and teratogenicity and developmental disorders are associated with mercury exposure (Harari et al. 2012; Karagas et al. 2012). In birds, reduced reproductive performance (Burger and Gochfeld 1997; Evers et al. 2008), impaired territorial fidelity and reduced incubation behaviour (Furness et al. 1986; Evers et al. 2008), a decreased body condition (Misztal-Szkudlinska et al. 2018) or mercury-related brain damage in bald eagles (a fish-eating species; Rutkiewicz et al. 2011) have been revealed.

As mentioned above, fish and other aquatic organisms are the main reservoirs of mercury (Kim et al. 2016). The content of mercury in fish bodies is highly variable and depends on many circumstances. Piscivorous birds, who are dependent on fish as the main source of their diet, are, thus, at high risk of mercury accumulation and the consequent negative health impacts (Nam et al. 2005). The mercury content in fish may reflect the contamination of the sampling site. Consequently, the exposure and effects on birds might be influenced by the location where the birds feed themselves (point sources). However, many of these birds are migratory and, thus, the concentrations and effects of mercury also vary over time. As predators, they can be used as good indicators of ecosystems’ spatial and temporal mercury contamination. Moreover, the levels of mercury are important indicators of an animal’s health (Furtado et al. 2019).

The aim of this study was to assess the total mercury content in the main detoxifying organs (liver and kidney) and in the muscles of adult great cormorants (*Phalacrocorax carbo)* caught at two localities in southern Poland. The aim was to compare the locality with the iron ore mining history (the Krogulna ponds) with a site without such a load (the Nysa Kłodzka River). The differences between mercury levels in the studied organs and also the effect of the location on the mercury content in the tissues were evaluated.

## MATERIAL AND METHODS

### Cormorant (*Phalacrocorax carbo*) sampling

A total of 45 adult great cormorants were shot down between October and December with permission of the Regional Directorate for Environmental Protection in Opole (Regionalna Dyrekcja Ochrony Środowiska, WPN.6401.18.2013.AP) during the monitoring of the effect of cormorants on local ichthyofauna. The cormorants were 2–3 years old (based on their size). Three tissues – liver, kidney and muscle were then obtained by the authors during the autopsy for the mercury analysis. A total of 135 samples were collected, placed into polyethylene bags, labelled and frozen (–22 °C) until the total mercury (THg) analysis was carried out.

### Mercury determination

The total mercury (THg) content in the tissues was determined by cold vapour atomic absorption spectrometry using an AMA 254 analyser (Altec Ltd., Dvur Kralove nad Labem, Czech Republic). The samples were thawed, weighed (approximately 50 mg), put into combustion boats and inserted into the AMA 254 without any sample preparation. The detection limit for THg is 1 μg/kg. The limit of detection (LOD) was set as the sum of triple the standard deviation of a blank and a blank mean value. The accuracy of the values for THg was validated using the standard reference material BCR-CRM 464 (Tuna Fish, IRMM, Belgium; declared value: 5.24 mg/kg; ascertained value: 4.77 ± 0.18 mg/kg). The total mercury concentration in the fish tissues is given in mg/kg fresh weight (f.w.).

### Study area

**Figure 1:**
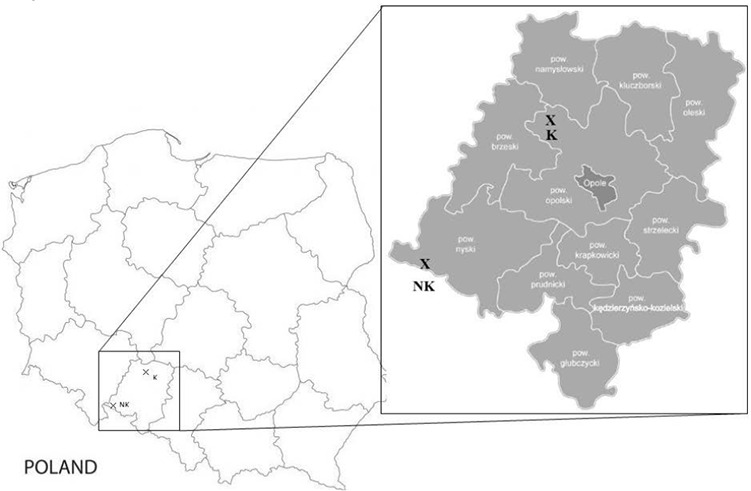
The map of the study area in Poland K = the Krogulna ponds; NK = the Nysa Kłodzka River

In this study, two different localities in Poland were monitored – the Krogulna ponds (K) and the Nysa Kłodzka River (NK) (Figure 1). The Krogulna ponds are located near the village of Krogulna, 40 km from Opole and 17 km from Namysłów and it is also a name of a fishing farm using traditional soil ponds for carp farming. At present, the farm is a part of the organisational structure of the Regionalna Dyrekcja Lasów Państwowych in Katowice. It operates on an area of 667 hectares of ponds, which are located in the southwestern part of the Opole Voivodeship. These ponds are grouped in eight fishing facilities, and 55 reservoirs fed by water mainly from the Stobrawa River basin. The main activity of the farm is breeding of freshwater fish, especially carp. Equally important, however, are measures to protect nature and to maintain the ponds as reservoirs for water retention (www.krogulna.pl). The shooting of great cormorants (*n* = 20) took place in the area of the farm. Krogulna is the locality with an iron ore mining history (the Krogulna ponds). The history of iron mining is related to Olesno, located about 50 km from Krogulna, in the upper reaches of the Stobrawa River. In the mid-nineteenth century, 12 iron ore mines (bog iron ore) as well as the developed iron and glass industries operated in the Olesno district. In 1858, the annual production of pig iron was 9 447 tonnes. Later, production gradually decreased and ended in the late 19^th^ century. The blast furnace slag heaps located near the banks of the Stobrawa River remained there for a long time after the former metallurgical production and, thus, may have represented a source of the local contamination.

The second locality is the Nysa Kłodzka River. Eastern Nysa, also known by the Polish name Nysa Kłodzka, is a river in southwestern Poland, a left tributary of the Odra River, with a length of 189 km and a catchment area of 4 570 km². It is situated behind the Nysa dam. The river is part of the region of Lower Silesia and the Opole Voivodeship. It is partially regulated, as the river has often left its banks in the past and flooded the surrounding towns, sometimes completely destroying them. The shooting of the great cormorants (*n* = 25) took place on the dam and in the vicinity of the river. The dam is managed by the Polish Angling Association and is used for sport fishing. This locality is considered to be free of mercury, as no point-source of mercury is known for that area, e.g., from chemical plants or other human activities. The locations are about 65 km far from each other (Figure 1).

### Statistical analysis

Unistat for Excel v6.5 was used for the statistical analyses and graphical presentations. First, the data were analysed to assess for normality (Shapiro-Wilk test). Due to the fact that the assumptions for the parametric analysis were not met, non-parametric tests were performed. The Mann-Whitney test was used for the comparison of the mercury content between the localities and the Kruskal-Wallis analysis of variance (ANOVA) followed by a post-hoc test (Tukey’s HSD) were used for the assessment of the mercury content differences between the organs from the great cormorants from one locality. The level of significance was set at *P* < 0.05.

## RESULTS

### The Krogulna ponds

**Figure 2:**
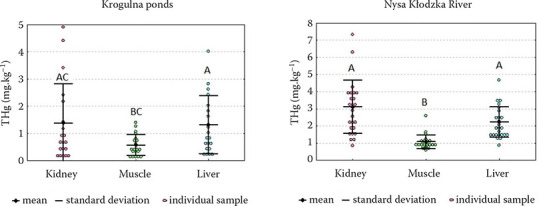
Total mercury content (mean ± SD) in the samples of the three tissues of the great cormorant from the Krogulna ponds and the Nysa Kłodzka River ^A,B^Indices indicate significant differences

The total mercury content in the great cormorant tissues, including the individual samples, is shown in Figure 2.

The average content of the total mercury assessed in the tissues of the great cormorant captured at the Krogulna ponds is presented in Table 1.

**Table 1:** The main statistical characteristics of the THg content in the tissues of the great cormorant from the monitored localities (mg/kg fresh weight)

Statistical characteristics	Liver			Kidney			Muscle	
	K	NK		K	NK		K	NK
Mean	1.33	2.28		1.39	3.12		0.58	1.09
Median	0.89	2.05		0.74	3.05		0.44	0.98
Variance	1.13	0.75		2.03	2.41		0.15	0.16
Standard deviation	1.06	0.87		1.43	1.55		0.38	0.40
Standard error	0.24	0.17		0.32	0.31		0.09	0.08
Coefficient of variation	0.80	0.38		1.03	0.50		0.66	0.37
Minimum	0.23	0.88		0.18	0.86		0.15	0.59
Maximum	4.02	4.67		4.90	7.32		1.40	2.60

K = the Krogulna ponds (*n* = 20); NK = the Nysa Kłodzka River (*n* = 25)

The lowest content (mean ± SD) was found in the muscle (0.58 ± 0.38 mg/kg), while it was higher in the liver (1.33 ± 1.06 mg/kg) and the highest in the kidney tissue (1.39 ± 1.42^ ^mg/kg). The highest individual value of the THg was found in the kidney (4.90 mg/kg), on the contrary, the lowest value was found in the muscle (0.15 mg/kg).

According to the obtained results, mercury cumulates mainly in the liver and kidney as the main detoxifying organs and the typical organs of metal storage (Saeki et al. 2000). The statistical evaluation did not find any significant difference between the liver and kidney at this locality (*P* = 0.893 2). After the comparison of the mercury content between the muscles and kidneys, again, no significant difference was found (*P* = 0.112 0). On the other hand, the liver and muscle mercury contents differed significantly (*P* = 0.037 6).

### Nysa Kłodzka River

The results of the total mercury content in the great cormorant tissues from this locality are shown in Figure 2.

The average content of the total mercury assessed in the tissues of the great cormorant captured at the Nysa Kłodzka River is presented in Table 1.

The lowest average value (mean ± SD) of the mercury content was again found in the cormorant muscle (1.09 ± 0.40 mg/kg) followed by the liver (2.28 ± 0.87 mg/kg), and the highest was detected in the kidney (3.12 ± 1.55 mg/kg) tissues. The highest individual mercury value was found in the kidney (7.32 mg/kg), while the lowest was found in the muscle (0.59 mg/kg), a pattern that resembled the Krogulna ponds.

When comparing the mercury content in the individual organs, it was not statistically significantly different between the liver and kidney (*P* = 0.330 0), while the muscle levels were significantly lower than both the kidney (*P* = 0.000 0) and liver levels (*P* = 0.000 0).

### Comparison of the localities

**Figure 3:**
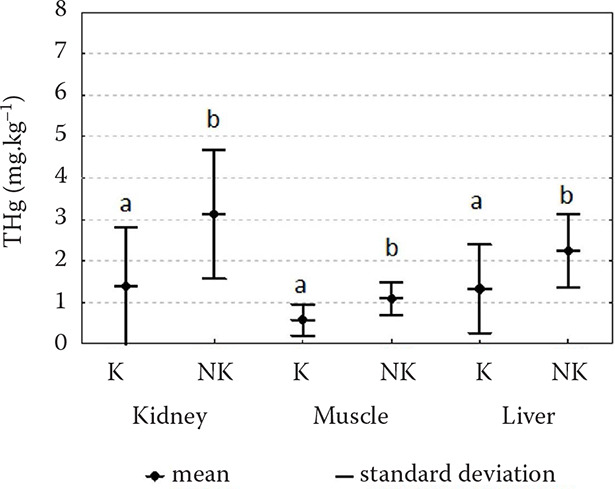
Comparison of the total mercury content in the individual organs between the monitored localities (mean ± SD) ^a,b^Indices indicate significant differences between the organs from the monitored localities K = the Krogulna ponds; NK = the Nysa Kłodzka River

The results for the total mercury content in the monitored organs for both localities are given in Table 1. The highest mercury value was found in the great cormorant kidneys from the Nysa Kłodzka River (7.32 mg/kg). When comparing the mercury content in the individual organs from both localities, it was found that the mercury content in both localities differed significantly for all the tissues (liver *P* = 0.001 8; muscle *P* = 0.000 2; kidney *P* = 0.000 2). The differences between the monitored localities are graphically presented in Figure 3.

## DISCUSSION

Wetlands and other aquatic ecosystems can be a place of Hg accumulation and conversion to methylmercury, which easily enters organisms and the food chain (Ma et al. 2017). Species at the top of aquatic food chains are highly susceptible to the effects of mercury. Piscivorous birds living in these areas are exposed to mercury mainly from the consumption of fish, which serve as its reservoir (Fournier et al. 2002). Cope et al. (1990) found that fish from acidic lakes with lower alkalinity may cumulate higher amounts of mercury than those from neutral-pH lakes, and this can influence birds feeding on the prey from such locations. The amount of mercury in the body also depends on the physiological mechanisms that regulate the uptake, distribution, and excretion of this substance. Birds eliminate mercury through faeces, feathers and eggs. Methylmercury has a high affinity for the sulfhydryl groups and is deposited into the keratin of feathers. Fully-grown feathers become physiologically isolated from the rest of the body and the further deposition of mercury into them as well as the efflux of mercury back to the body are impossible. Mercury levels in the body organs might, therefore, be relatively low during feather growth and increase once the feathers are fully-grown (Fournier et al. 2002). Moreover, some studies have documented changes in the mercury body burden before and after moulting in adult birds (Braune 1987). Cormorants in the northern hemisphere moult a large portion of their feathers (except for the head and tail) in the period of June to December each year (Saeki et al. 2000). In this study, great cormorants were caught between October and December at both locations which are of the same climate, so we do not expect any significant difference in their moulting.

Great cormorants come to Poland to breed at the end of February and the beginning of March. Each year they spend 7–8 months in the breeding areas (Fabczak et al. 2003), which means that the load of the locations they live on has an important impact on the cumulation of Hg in their food and consequently on the health of cormorants. They especially feed on older and larger fish with an average length of 20–25 cm (Houserova et al. 2007) and are known to accumulate high levels of several contaminants which makes them useful bioindicators, also due to their wide geographic range and their long lifespan (Skoric et al. 2012). They have been reported to have high deposit levels of mercury in their tissues. Several studies focused on the mercury content in feathers (Ochoa-Acuna et al. 2002; Nam et al. 2005; Malik and Zeb 2009; Misztal-Szkudlinska et al. 2012; Lavoie et al. 2015), but heavy metals in the feathers can also be of exogenous origin (superficial contamination) and are also influenced by the type of feathers and the age of the bird, which has to be taken into consideration when interpreting data (Skoric et al. 2012; Borghesi et al. 2016).

Many other studies also measured the metal content in internal organs. The total Hg concentrations in the organs are of high informative value as cormorants partially demethylate organic Hg in the tissues, such as the liver and kidneys and store a large portion of their Hg burdens in an inorganic form (Saeki et al. 2000). The main tissues studied are the liver, kidneys, muscle, intestines or bones. A high total mercury content in the liver (Rajaei et al. 2011; Skoric et al. 2012) or the liver and kidneys is frequently reported (Houserova et al. 2005; Misztal-Szkudlinska et al. 2011).

In this study, which is aimed at the monitoring of the mercury levels at two locations in Poland where the great cormorants live, at the Krogulna ponds, both the liver and kidney mercury contents (Table 1) were lower than the levels found in the work by Kral et al. (2017), Houserova et al. (2005) and Houserova et al. (2007) in the Czech Republic. On the other hand, these values resemble those (when calculated from the dry weight (d.w.) to fresh weight (f.w.) found by Skoric et al. (2012) in Serbia in the liver of younger birds, Alomar et al. (2016) in France, Goutner et al. (2011) in Greece or Lehel et al. (2013) in Hungary (Table 2).

**Table 2:** Total mercury content in the great cormorant from the different studies, mean ± SD

Tissue	Value	Unit	Study/country
Liver – Krogulna	1.33 ± 1.06	mg/kg f.w.	present study/Poland
Kidney – Krogulna	1.39 ± 1.42		
Muscle – Krogulna	0.58 ± 0.09		
Liver – Nysa Kłodzka	2.28 ± 0.87		
Kidney – Nysa Kłodzka	3.12 ± 1.55		
Muscle – Nysa Kłodzka	1.09 ± 0.40		

Liver – adult	36.24	mg/kg d.w. (only median in this study)	Houserova et al. (2005)/Czech Republic
Kidney – adult	7.61		
Muscle – adult	3.34		
Liver – juvenile	5.77		
Kidney – juvenile	4.61		
Muscle – juvenile	2.59		

Liver – adult	42.20 ± 6.28	mg/kg d.w.	Houserova et al. (2007)/Czech Republic
Kidney – adult	7.20 ± 1.00		
Muscle – adult	3.40 ± 0.37		
Liver – young	7.50 ± 1.63		
Kidney – young	4.10 ± 0.49		
Muscle – young	2.50 ± 0.33		

Liver	2.12 ± 0.22	mg/kg f.w.	Kral et al. (2017)/Czech Republic
Kidney	2.23 ± 0.30		
Muscle	0.60 ± 0.06		

Liver	3.39 ± 1.39	μg/kg d.w.	Saeki et al. (2000)/Japan
Kidney	4.05 ± 2.18		
Muscle	1.27 ± 0.35		

Liver – Biwa (Japan)	3.40 ± 1.10	μg/kg d.w.	Nam et al. (2005)/Japan
Liver – Mie (Japan)	7.40 ± 6.70		
Kidney – Biwa (Japan)	2.50 ± 2.20		
Kidney – Mie (Japan)	7.10 ± 8.90		
Muscle – Biwa (Japan)	1.20 ± 0.60		
Muscle – Mie (Japan)	1.40 ± 0.90		

Liver	8.32 ± 1.32	mg/kg (originally ppm – d.w. or f.w. not mentioned)	Mazloomi et al. (2008)/Iran
Kidney	9.25 ± 1.71		
Muscle	2.06 ± 0.22		

Liver	8 089.60	ng/g d.w.	Goutner et al. (2011)/Greece

Liver – adult	3.18 ±1.11	μg/g d.w.	Skoric et al. (2012)/Serbia
Muscle – adult	1.65 ± 0.58		
Liver – subadult	6.14 ± 2.51		
Muscle – subadult	2.86 ± 1.28		
Liver – juvenile	6.18 ± 2.21		
Muscle – juvenile	2.84 ± 0.59		

Liver	30.67 ± 14.08	μg/g d.w.	Hribsek et al. (2017)/Serbia
Kidney	17.41 ± 5.68		
Muscle	9.48 ± 1.17		

Kidney	8.95 ± 1.23	mg/kg d.w.	Kitowski et al. (2015)/Poland

Liver – adult	15.51 ± 17.30	μg/g d.w.	Misztal-Szkudlinska et al. (2011)/Poland
Kidney – adult	30.21 ± 47.93		
Muscle – adult	2.15 ± 1.10		
Liver – juvenile	10.96 ± 11.42		
Kidney – juvenile	17.58 ± 22.96		
Muscle – juvenile	1.61 ± 0.82		

Liver	14.60 ± 16.30	μg/g d.w.	Misztal-Szkudlinska et al. (2018)/Poland
Kidney	27.70 ± 44.20		
Muscle	2.05 ± 1.07		

Liver	5.47 ± 4.76	mg/kg d.w.	Alomar et al. (2016)/France

Liver	5.65 ± 3.62	μg/g f.w.	Albertos et al. (2020)/Spain
Kidney	4.40 ± 4.75		

Liver – adult	4.48 ± 3.34	mg/kg d.w.	Lehel et al. (2013)/Hungary
Liver – juvenile	2.68 ± 2.09		

Muscle	1.00 ± 0.40	μg/g d.w.	Lehel et al. (2022)/Hungary

The mean water content in the tissues and organs analysed is ca 75% (between 70–76%) according to Misztal-Szkudlinska et al. (2018). The dry matter is thus approximately 25% (1/4 of the sample weight). Thus, the approximate calculations are d.w. = f.w. × 4 and f.w. = d.w./4 in case of the same units

d.w. = dry weight; f.w. = fresh weight

The values found in this study for the Nysa Kłodzka River for the liver and kidneys (Table 1) are similar to Kitowski et al. (2015) in Poland and to Kral et al. (2017) in the Czech Republic (Table 2). When compared with the mercury burden found in cormorants from other European localities, the results are lower than those found by Houserova et al. (2005) and Houserova et al. (2007) in the Czech Republic, Misztal-Szkudlinska et al. (2011) and Misztal-Szkudlinska et al. (2018) in Poland, Albertos et al. (2020) in Spain and Hribsek et al. (2017) for the Danube River area in Serbia. Our results were, on the contrary, higher than those found by Lehel et al. (2013) and Lehel et al. (2022) in Hungary (Table 2).

All the locations in Europe are considered free from a known strong local source of mercury contamination.

The mercury content in the cormorants in this study decreased as follows: kidneys > liver > muscle. The same conclusions were reached by Saeki et al. (2000), Mazloomi et al. (2008), Misztal-Szkudlinska et al. (2011), Kral et al. (2017) and Misztal-Szkudlinska et al. (2018). On the other hand, Nam et al. (2005), Houserova et al. (2005), Houserova et al. (2007), Hribsek et al. (2017) and Albertos et al. (2020) order the tissues according to their decreasing total mercury content in different succession: liver > kidney > muscle. In all the cases, the muscle is found to contain significantly lower concentrations of mercury than the liver and/or kidneys.

Misztal-Szkudlinska et al. (2011) and Robinson et al. (2012) revealed that females of piscivores have lower levels of mercury in their bodies than males, one of the reasons for it being that females excrete mercury when laying eggs. However, embryos are particularly sensitive to mercury toxicity and, therefore, it would be maladaptive to dump fatal quantities of mercury into the eggs, so this mechanism does not have to decrease the levels in females substantially. Metabolic differences between male and female birds are not fully understood yet as well as the influence of a parasite infestation on the mercury levels in the body (Robinson et al. 2010). On the other hand, Misztal-Szkudlinska et al. (2018) found no differences in the mercury levels between the sexes. The birds in this study were not sampled during the nesting season, this is why this study did not focus on a sex role in the mercury cumulation.

Age, on the other hand, has a very important impact. Juvenile and subadult cormorants cumulate more mercury in their tissues according to Skoric et al. (2012). Contrary to this, Saeki et al. (2000), Houserova et al. (2005), Houserova et al. (2007), Mazloomi et al. (2008), Misztal-Szkudlinska et al. (2011) and Lehel et al. (2013) describe the higher accumulation of mercury in adult birds compared to juvenile or chick specimens. Moreover, in the study of Misztal-Szkudlinska et al. (2018), there were no differences found between juveniles and adults. Juvenile cormorants were not sampled in this study, but for the evaluation of the effect on the whole cormorant population’s contamination and health in southern Poland, age differences should be evaluated and elucidated for these localities during the next research phase.

In the Krogulna ponds, a location with an iron ore mining history, the total mercury content in all the studied organs is significantly lower than in the Nysa Kłodzka River (Figure 3). In contrast, although the iron production in the Stobrawa River catchment supplying the ponds at the Krogulna ended over 100 years ago, the level of lead in the tissues of the cormorants feeding there is 2 to 4 times higher for the muscles and livers, respectively, than in the Nysa Kłodzka River (Krogulna: 0.049 μg/g f.w. and 0.224 μg/g f.w. versus the Nysa Kłodzka River: 0.025 μg/g f.w. and 0.063 μg/g f.w., respectively; Spodniewska et al. 2016). The reason for this finding is unclear and further analyses of mercury and lead levels in the fish from both locations and the sediments from Krogulna are necessary as there are no data on them available. The mercury and lead concentration in the sediments of the Otmuchów Reservoir located on the Nysa Kłodzka River were analysed by Rzetala (2016) and the average values are 0.07 mg/kg and 62.00 mg/kg, respectively. The Nysa Kłodzka River sediments were analysed also by Lis and Pasieczna (1995) and the measured average values were 0.08 μg/kg for mercury and 57.00 mg/kg for lead.

The explanation of the unexpected differences in the total mercury content might be due to the different diet. The work of Martyniak et al. (2013) shows that the cormorants in the Krogulna ponds locality have a relatively monotonous and stable diet. It consists mainly of carp from the fishing farm. In this locality, the cormorants are exposed to a lower mercury content, as there is no excessive accumulation of this metal described in omnivorous carp (Barus and Oliva 1995; Kottelat and Freyhof 2007). This is due to the position of the carp in the food chain and its food habits, where the carp feed mainly on plankton (Barus and Oliva 1995). In contrast, in the Nysa Kłodzka River, the cormorants have a much-varied diet. The cormorants can catch both predatory and non-predatory fish and Martyniak et al. (2013) stated that a diverse range of fish species was found in the digestive tract of cormorants from this locality, mainly benthic-feeding bream, and the predatory fish perch and pikeperch. The cormorants in this area are, thus, exposed to different (higher) levels of mercury in the fish they feed on. Also, Ackerman et al. (2016) reported that the mercury concentrations in the birds’ blood vary between species with different diets, and birds that feed on fish of higher trophic levels have higher mercury concentrations due to mercury biomagnification in the food chain, which is consistent with our results.

Several studies indicate that the levels of liver mercury in the cormorant above 4 μg/g d.w. (approx. 1 μg/g f.w.) can have negative effects on their growth, reproduction, development, haematology, and metabolism (Goutner et al. 2011; Skoric et al. 2012). In our study, the values for the Krogulna ponds were slightly above this value and even higher than this value for the Nysa Kłodzka River, so the possible negative long-term impact on their health is not excluded and should be further evaluated.

The total mercury content in the great cormorants from this study decreased as follows: kidneys > liver > muscle in both monitored localities. The amount of mercury in the individual organs varies significantly between the localities. The assumption that birds from the Krogulna ponds, which were created in an area where iron ore mining activities took place in the past, should have a higher total mercury content in the cormorant organs was not confirmed, as the great cormorants from the Nysa Kłodzka River, the location without a mining load, had significantly higher mercury levels in their organs compared to the Krogulna ponds. The accumulation of mercury is, therefore, dependent on the different fish stocks in these two localities (omnivorous fish in Krogulna ponds versus predatory fish in the Nysa Kłodzka River), and also due to the different types of prevailing food, which leads to a different level of the accumulation of mercury in the great cormorant organisms. An analysis of the mercury levels in the fish and sediments from the respective locations would be also beneficial to exclude any unknown contamination in the localities, and the pH and alkalinity of the water could also be assessed to evaluate any possible differences in the mercury bioavailability to the food chain in both localities. Additional studies to assess patterns of seasonal changes as well as those monitoring the health effects of mercury cumulation on juveniles should be performed.
